# Scaling up a decentralized offline patient ID generation and matching algorithm to accelerate universal health coverage: Insights from a literature review and health facility survey in Nigeria

**DOI:** 10.3389/fdgth.2022.985337

**Published:** 2022-09-07

**Authors:** Emeka Chukwu, Iniobong Ekong, Lalit Garg

**Affiliations:** ^1^ Department of Computer Information Systems, Faculty of Information Communication Technology, University of Malta, Msida, Malta; ^2^ Department of Health Planning Research and Statistics (HPRS), Health and Human Services Secretariat, FCT, Abuja, Nigeria

**Keywords:** client registry, Master Patient Index (MPI), Universal Patient Identifier (UPI), decentralised identifier, patient matching, interoperability, health information exchange, digital health

## Abstract

**Background:**

Quality of health service delivery data remains sub-optimal in many Low and middle-income countries (LMICs) despite over a decade of progress in digitization and Health Management Information Systems (HMIS) improvements. Identifying everyone residing in a country utilizing universal civil registration and/or national unique identification number systems especially for vulnerable patients seeking care within the care continuum is an essential part of pursuing universal health coverage (UHC). Many different strategies or candidate digital technologies exist for uniquely identifying and tracking patients within a health system, and the different strategies also have their advantages and trade-offs. The recent approval of Decentralized identifier (DID) core specification by World Wide Web Consortium (W3C) heralds the search for consensus on standard interoperable DID methods.

**Objective:**

This paper aims to: (1) assess how candidate Patient Identification Systems fit the digital Patient ID desirable attributes framework in literature; and (2) use insights from Nigeria to propose the scale-up of an offline, interoperable decentralized Patient ID generation and a matching model for addressing network reliability challenges of centralized electronic registries in LMICs.

**Methods:**

We combined: (i) systematic review of the literature to identify the characteristics of leading candidates for Patient ID systems, with (ii) review of policies and (iii) quantitative survey of 14 general hospitals in Nigeria's Federal Capital Territory to understand the model(s) of patient ID strategies currently implemented by public hospitals.

**Results:**

Evidence from the literature review and quantitative survey showed that no current Patient ID strategy in Nigeria simultaneously meets the six attributes of uniqueness, unchanging, uncontroversial, inexpensive, ubiquitous, and uncomplicated required for ensuring the reliability of unique patient identification systems and of the HMIS more generally.

**Conclusions:**

The findings are used to propose a model of algorithms for universal-offline Patient ID generation and matching models that is cost effective and can be easily scaled-up throughout Nigeria. The prototype has promise for generating and validating a universally unique Patient ID given a set of patient characteristics without a central rigid authority. The model can also help to fast-track the implementation of a Master Patient Index (MPI) and interoperability of existing digital health platforms in LMICs.

## Introduction

### Global digital health

Efforts to support the global digitization of health systems by United Nations (UN) member states is well documented in 2005, 2013, and 2018 World health Assembly resolutions (1, 2). The World Health Organization (WHO) and the United States Agency for International Development (USAID) and other development partners recently developed strategies for digital health and digitization (3, 4). The role of a unique identifier such as avoiding duplicates and improving quality has been well established (5). The Luxemburgish health authorities shared their five years' experience designing a Master Patient Index (MPI) between 2014 and 2018, illustrating steps and complexity of the undertaking (6). Different states in the US also use MPI at different degrees (7). Master Patient Index (MPI) has been experimented in Argentina (8), Myanmar (9), and Open source software has been developed for MPI (10).

### Nigeria context

In 2016, the Nigerian government developed and launched a national digital health strategy which is now under review to systematically adopt digital technologies in healthcare (11). The strategy considered the many different components necessary to enable an integrated health system including unique digital identifier for delivering Universal Health Coverage (UHC) (12) and to deliver Universal Health Coverage (UHC) . Sub-regional governments like the Federal Capital Territory Health and Human Services Secretariat also developed mirror strategies geared towards using digital technologies to drive UHC (13). Health systems planning and programming in Nigeria, like most developing countries, still heavily rely on multi-year surveys (14), despite the many investments and progress in the collection, curation, and use of service delivery data using its National Health Management Information System (NHMIS) (15). For over a decade, this routine health information system has aggregated service delivery data from health facilities and communities using the District Health Information System version two (DHIS2) web portal. This web portal aggregates and serves as the repository for healthcare data in over 60 countries in developing countries (16). Poor quality of data in the DHIS2 web portal has been cited as one of the reasons for reliance on multi-year surveys for strategic level decision-making. One WHO-based quality assessment found that health facility reported data could be incomplete for as high as 40 percent of the time (17). In addition, up to 60 percent of “events” in the health facility register were under-reported, amongst other inconsistencies (17). Aggregate service delivery data are mostly captured on the DHIS2 portal using monthly summary forms from health facility paper registers. The paper summary forms are sent to the Local Government Authority (LGA) Monitoring and Evaluation (M&E) officers, who capture them in the DHIS2 web portal. Each of the 774 local governments in Nigeria helps capture the 40,300 health facilities' data every month (15, 18). The health facilities in Nigeria are described as publicly or privately owned and further categorized as Primary, Secondary, or Tertiary health facilities according to the type of service they provide. In Nigeria, 90 percent of these health facilities are Primary Health Care (PHC) comprising of PHC Centers and PHC Clinics.

The Federal Capital Territory (FCT) Abuja in Nigeria was selected for this novel study because of the huge level of government investment in Information and Communication Technologies (ICT) for health as well as recent development in patient identification systems in public secondary hospitals in the Territory. The study was however restricted to the FCT only due to resource limitations and the short deadline for submission to this special collection on scale-up and sustainability of digital health interventions in LMICs.

### Shared digital health records

Digital individualized healthcare data management and reporting will boost HMIS data quality at all healthcare levels through auditability and linkability (19). Patients are seen longitudinally over time in a continuum of care. Sometimes data sources for a Patient's information vary. Data about a Patient may be in different formats across health domains, departments, institutions, or software-vendor systems. A patient's digital health record may be managed using an Electronic Medical Record (EMR) in a typical health facility. An EMR will have the Patient's medical history, laboratory investigations and results, medications, and many more. When a Patient's health record is fragmented across institutions, software systems, departments, then a complete picture of their record is only possible when these records are linked. Linked Patient records can help reduce duplicates which can affect efficient resource allocation and utilization. Appropriate identification and record linkage can help reduce harm and allow for better aggregation of linked records (20). Fellegi et al. in 1969 were one of the earlier pioneers, and they proposed record linkage to identify duplicates (21). Patient misidentification is a primary cause of Patient harm in an EMR (22). To our knowledge, no linked health records currently contribute to the NHMIS repository in Nigeria. We also did not find other evidence from other low and middle-income countries. Most health institutions in Nigeria use institution-specific Patient ID generation and matching, though an ideal Patient identifier should positively identify a patient, protect their privacy, and be cost-effective. Despite the increasing adoption of EMR systems in Nigeria, there is currently no centrally accessible electronic patient database that uniquely identifies patients, stores patient demographics and allows personal health records to be shared seamlessly and cost-effectively. It is cost prohibitive to build and implement the required ICT infrastructure and interoperability standards for a centralized electronic registry coupled with the inadequate funding for digital health, digital divide and network connectivity challenges. A decentralized interoperable patient registry system will serve as a cost effective, foundational approach to implementing interoperability standards for health information exchange in Nigeria and accelerating the establishment of a futuristic centralized patient registry or MPI. This will ensure meaningful use of existing digital health applications by promoting health data gathering, enhancing care coordination and exchange of patient information which in turn will lead to improved health outcomes.

### Country unique health identifier strategy

Countries can be grouped into five categories based on the strategy adopted. First, some countries assign a national unique health identifier (UHI) to each person for healthcare, in addition to allocating a unique identification number (UIN) to each individual *via* a national identity management system (23). Second, other countries only use the UIN for health purposes without the need for creating an alternative unique number specifically for health. Third, in yet other countries, individuals are assigned a UHI without having a UIN. Fourth, some low- and middle-income countries (LMICs) have neither the UIN or the UHI at the national level, and different health facilities generate their own patient numbers for administrative purposes but with limited utility for linking data with other health systems. Finally, some LMICs have UIN at national level, do not use it for health purposes and different health facilities assign disparate patient IDs. UHI will no doubt help LMICs avoid duplication in the counting of key populations attending health services and increase uptake of critical services by eliminating stigmatization through a confidential service recognition system that uniquely identifies individual without disclosing personal information. It will also help to improve quality of care by providing longitudinal record of patients interaction with the health care system throughout the care continuum and help track patients who have missed referrals or are lost to follow up.

### Study objective

This paper aimed to systematically review the literature of patient identifier schemes and discuss their trade-offs and use the insights to propose a prototype algorithm for Patient identifiers and matching supported with decentralized identifiers. and uses lessons learned to propose the scale-up of an offline decentralized Patient ID generation and matching model with the potential for addressing network reliability challenges in LMICs.

## Methodology

### Study setting

The *current population* of *Nigeria* is 218.6 million people based on projections of the latest United Nations data, with 51.2% of these residing in urban areas (24). The Federal Capital Territory (FCT) Abuja which is the Nation's Capital city is located between latitudes 8◦25′ and 9◦ 25′ north of the equator and longitudes 6◦45′ and 7◦ 45′ east of Greenwich. The FCT covers a land area of 8,000 square kilometres with an estimated population of 3.6 Million in 2016 (25). The territory is made up of six area councils which corresponds to the local government areas in other states of the Federation that supervises and funds the PHC facilities as well as have overall responsibility for this level of health care service delivery in Nigeria. The area councils are namely: Abuja Municipal, Kwali, Bwari, Gwagwalada, Kuje and Abaji.

### Study design

The study combined: (i) a review of the literature on existing “healthcare facility Patient ID schemes”; with (ii) documents analysis of current national, functional, institutional patient identifiers across Nigeria's sectors (18), and (iii) a Google questionnaire survey administered to all 14 public secondary health facilities in the FCT to understand the Patient ID systems adopted by health facilities.

### Data collection and analysis

We reviewed the literature of existing “healthcare facility Patient ID schemes” using systematic search on select public health databases. Given that digital health is the intersection of two fields, Information Communication Technology (ICT) and Health, we chose the two most popular scholarly databases in both fields. The IEEExplore and the PubMed databases, the search was augmented with traditional google search. In the IEEEXplore settings, the listed search terms in Table 1 were searched in title, abstract and metadata settings. In the PubMed advanced setting, the search query was set to search the title and abstract. We limited and excluded the use of the keyword MPI as it has other meaning in clinical science returning over 28,000 results. Similarly, the use of identifier was not used for the same reason.

**Table 1:** Summary of results from scholarly data base searches.

DATABASE/Keywords	IEEEXplore	PubMed
client AND registry	56	130
Master Patient Index	19	24
Universal Patient Identifier	5	3
TOTAL	80	157

**Figure 1:**
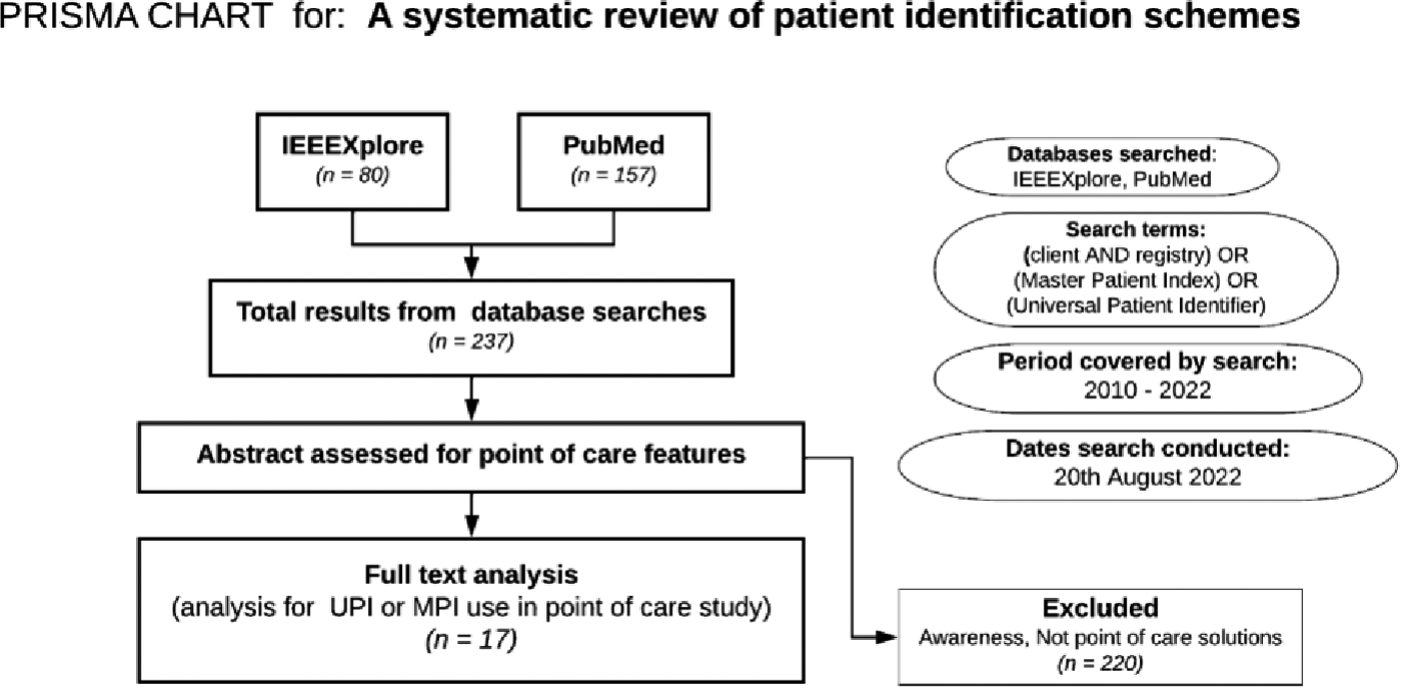
PRISMA for literature search approach for determining candidates for patient ID systems.

For relevance, the search period was limited to recent publications for the period 2010 and 2022. The systematic search was concluded and documented in August 2022. See the Preferred Reporting Items for Systematic Reviews and Meta-Analyses in Figure 1. We then reviewed current national, functional, institutional, and other forms of Identifiers across Nigeria's sectors (26). This was combined with authors’ expert knowledge as digital health thought leaders in the country.

In addition, findings from the literature review were used to inform the design of the online questionnaire survey distributed over a 1-month-period from 6 June to 5 July 2022. The questionnaires asked five key questions that sought to understand:

1. How health facilities generated/assigned patient ID: manual, computer generated or other.
2. The format of patient ID: numbers, letters, combinations of numbers and letters
3. Number of digits that patient ID compised of: <5, 5–10, or >10 digits
4. Whether patient IDs were serially or randomly generated
5. Other information captured by patient ID: Phone №, National ID №, etc.

The structured questionnaires were administered to all the 14 public secondary health facilities in the FCT on Patient ID systems adopted by each hospital. Only 11 of the 14 sary health facilities completed and returned the quantitative questionnaires. Quantitative data were exported from Google forms and processed Microsoft excel. Responses to the above questions were analysed using simple descriptive analysis because of the small sample size (*n* = 11) of the health facilities surveyed. The questionnaire used for the survey is attached as Supplementary Material S2. Findings from the literature review, document analysis of policies and the Google survey were then triangulated with authors' expert knowledge to propose a phone number-based deterministic Patient matching model. The model was extended using a probabilistic Patient matching model of patients’ demographic characteristics.

## Result

This section is presented in four parts – (1) the strategies and leading candidates for unique Patient ID. (2) Options for patient matching when unique identifiers are not possible. (3) Emerging Decentralized ID (4) Our model for Patient ID generation and matching.

### Strategies and leading candidates for unique patient identifiers

One strategy for managing a Patient's unique identifier is to use a central repository, sometimes referred to as Master Patient Index (MPI) or Client Registry (CR). In different jurisdictions, it can be either a national ID scheme, the health institution-managed scheme, a Master patient Index (MPI), or other functional IDs (23). An effective Patients’ ID scheme must consider the questions: How will the patients enroll? How does the Patient authenticate? What is the security of storage? How is the stored data governed? What are the trust mechanisms when governance is decentralized? What is the process for managing duplicates? How is the ID created and issued? Are there other social determinants? (23). McFarlene et al. captured six competing characteristics that an ideal Patient ID needs to meet: (1) Unique, (2) Unchanging, (3) Ubiquitous, (4) Uncomplicated, (5) Inexpensive, (6) Uncontroversial (27).

Similarly, patient identification is computer generated in some instances using a combination of attributes in Uganda (28), Bangladesh (29), and Burkina Faso (30). Resident card number has been used as Master Patient Index (MPI) in China (31). Some health institutions in the US attempted using Social Security Number (SSN) (23), Others use enterprise Master Patient Index (EMPI) (32). The promulgation of a Unique patient identifier legislation was blocked by congress in the US (33). Similarly, Electrocardiogram (ECG) signals have been used to encode unique signatures to identify an individual patient uniquely (34). Other biometrics like finger print in Nigeria (35) and Iris biometric identification in Kenya (36), Biometric patient identification and management (37). System generated Patient ID in is used in India (38). Systems for multiple patient identifiers have also been used (39). Bar-codes have been used with Open Data Kit (ODK) in Kisumu county, Kenya for HIV program (40).

**Figure 2:**
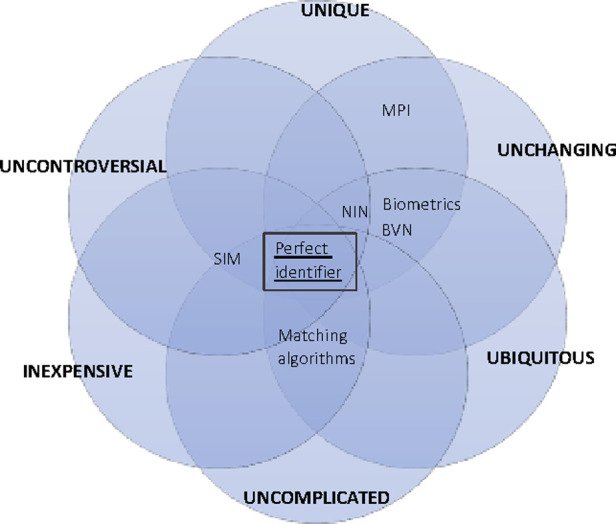
Framework for illustrating desirable attributes and trade-offs of patient ID.

Nigeria uses a National Identity Number (NIN) as her national ID number centrally managed by National Identity Management Commission (NIMC). Enrolment happens at designated National Identity Management Commission's (NIMC) enrolment centers. Though efforts are ongoing to enroll NIN at health institutions using custom made hardware. The Deposit Money Banks (DMO) in Nigeria use a parallel functional ID for service provision and authentication, know as Bank Verification Number (BVN). It is believed that governance and trust challenges influenced the creation of the BVN in 2014 despite NIN being the statutory national ID with a four-decade mandate and 13-year-old enabling law (41, 42). Some proponents suggest that telecommunications providers’ Subscriber Identification Module (SIM) number can be used as a functional ID for Patient identification. Some health institutions also use one unchanging biometric identifier to identify patients within their health institution uniquely. Nigeria has not implemented MPI for centralized Patient management. Given the current electricity, network, and computing infrastructure deficiencies, an MPI may not be feasible in Nigeria at this time. In addition, the decentralized governance structure with increasing state and sub-regional autonomies demarkets centralization inherent in traditional MPIs. Moreso, health is on the concurrent legislative list allowing states the leverage to create their own systems. Currently, health institutions in Nigeria manage their patient IDs. The desirable attributes and the possible candidates for Patient identification in Nigeria and where they fall are illustrated in Figure 2.

#### FCT health facility identification schemes

Nigeria's FCT has 14 public General Hospitals spread across its six Area Councils with each of these facilities managing their patient identifiers independently though with similar ID schemes. The Patient's biodata predominantly documented at the first visit include name, sex, date of birth, phone number, email and physical address as well as name, address and phone number of next of kin.

In addition to these biodata, hospital numbers are assigned to all patients during their first visit and recalled in subsequent visits. Our survey findings showed that patient IDs assigned in 90.9% of the facilities were serial numbers while 9.1% assigned the IDs randomly. Majority (63.6%) of these IDs were numbers while 36.4% were alphanumeric with hospital abbreviations added as prefixes to these numbers. 90% of the IDs had 5 or more digits while 5% had less than five digits.

EMRs deployment by two different vendors in eight of the 14 General hospitals has further optimized the patient ID scheme by adding any of driver's license number, international passport number or voters card number as additional fields to enable validation. In line with the requirements for mandatory use of the national identification number by the National Identity Management Commission (43), the National Identification Number (NIN) is mandatorily captured but not compulsory fields as most patients are yet to be enrolled. Then patient photograph is also captured using the webcam, after which a serialized hospital number is automatically generated. Though the software systems have the capability for fingerprint biometric capture, this is not yet activated. Patients' hospital numbers are initially searched and validated after a follow-up visit with a phone number, date of birth, or stored photograph. In the event of misplacement of hospital number, the Patient's name and/or phone number and/or date of birth is searched for and the patient record retrieved.

**Figure 3:**
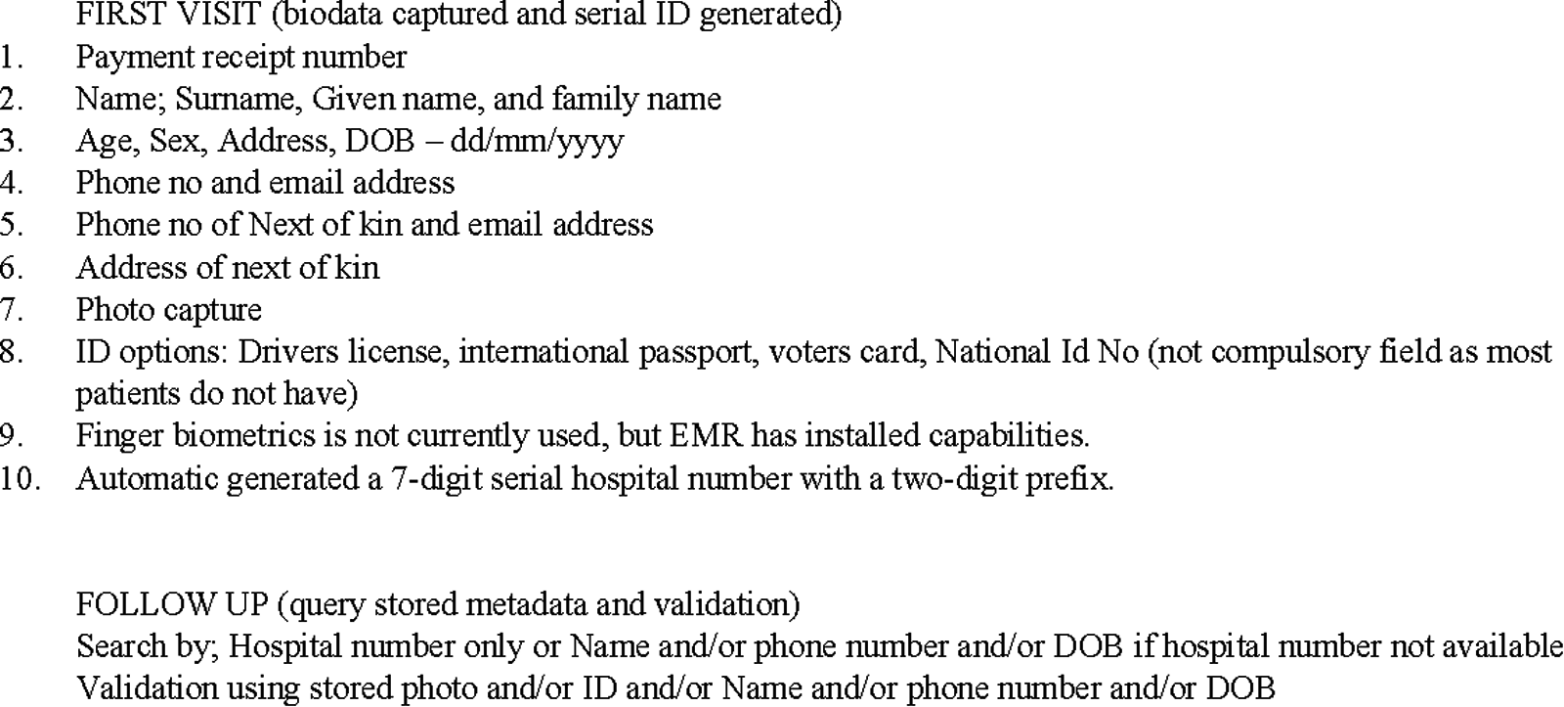
Patient EMR ID scheme in a typical FCT hospital.

In spite of the introduction of these EMR systems, it is not yet possible to link longitudinal patient records throughout the care continuum to allow for continuity of care and quality management in linewith UHC principles. Figure 3 illustrates the current patient ID scheme in a typical FCT EMR using a hospital.

### Options for patient matching when unique identification is not possible (matching algorithms)

We have established that there is currently no perfect ID scheme among the leading contenders to uniquely identify a patient's shared health record for many reasons. See Figure 1. Estonia, for instance, successfully implemented a centralized national ID for health identification (44). Investment, safety, trust, and security requirements for such cross-organization creation and authentication are higher than is currently possible for LMIC health facility infrastructure and information structures. An MPI facilitated Patient matching algorithms will in this case be critical in determining if a record exists in a shared repository or not. Checks like these can be used for efficient-create, retrieve-and-update operations, and duplicate management. Traditional MPIs make extensive use of matching algorithms for de-duplication.

Client Registries are used as controllers for health institution-specific identities associated with a patient (45). Incoming data “create” or “update” operations are matched for linkage with existing patient records. In “create”, if a positive patient match is not made, a new patient record is created in the MPI. The leading MPI mapping movement is based on HL7 Fast Healthcare Interoperability Resource (FHIR) OperationDefination resource “$match”. An MPI service recommends it for matching patient information stored in multiple databases (46). The implementation URL for this operation will be “*[base]/Patient/$match*”. The $match operation takes the patient “resource” with match attributes and the maximum number of returned records “count”. The $match operation will return an “operationsOutcome” FHIR resource, Patient resources representing possible matches, and outcome status codes. MPI-based ID creation and authentication require real-time connectivity to the centrally managed authentication infrastructure (or intermediary).

Three underlying mechanisms are broadly used for Patient matching – (1) deterministic, (2) probabilistic, and (3) deterministic + probabilistic (20). Deterministic matching is considered the most popular kind of matching algorithm because it uses exact unique and discriminatory identifiers (e.g., NIN, BVN, Phone number) for matching with only two possible results: positive-match or negative-match. Probabilistic matching, on the other hand, gives only the probability of match as an output. The most basic form of probabilistic matching is the average probability of a match for a pair of patient record attributes (45). The probabilistic technique can be classified into a simple fuzzy logic and statistical approach (often used in machine learning techniques) (47). Probabilistic matching algorithms can use the Patient's characteristics (like name, address, NIN, BVN, or phone number) for determining if a patient is true-match, false-match, true-nonmatch, or false-nonmatch from a list of matched records (27). In practice, patient matching algorithms use deterministic first, and when it fails, use probabilistic matching. This process remains the same even for jurisdictions like the UK with universal unique Patient identifiers (48). This problem is even more pronounced in the US. A recent study of 398,999 patient records shows that social security numbers recorded the second most frequent matching mismatch of 53.54 percent of duplicate pairs (49). Also, a report for the Office of National Coordinator Health IT in the US indicated that while positive patient match neared 90% within health institutions, they drop when matched against records from other institutions, even if the institution used the same EMR vendor (50).

### Emerging trend-decentralized ID

**Figure 4:**
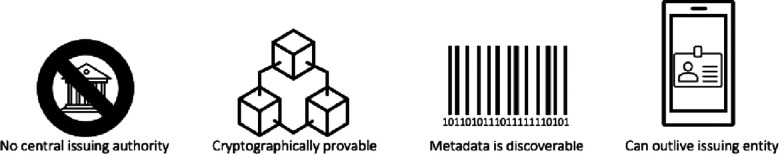
Characteristics of a decentralized ID scheme.

A new wave of research and discussions on decentralized ID schemes is championed by the World Wide Web Consortium (W3C) standards group (51). The W3C published a draft standard for Decentralized Identifiers (DIDs), a new form of identifiers (ID) capable of being validated without needing a central registry, identity provider, or digital certificate issuing authority. The draft standard which has just recently been approved sets the following criteria to classify an ID scheme as decentralized – the requirements are as set out in Figure 4.

Our decentralized patient ID generation and matching model leverages these characteristics while ensuring it meets optimal desired characteristics illustrated in Figure 2.

### Our model

Our model use case is a two-health facility scenario where both health facilities have intermittent internet connections to an EHR (which can also be a distributed Blockchain network). In this model, we extend the current FCT standard health facility Patient ID management schemes using a two self-contained step process:

1. Phone number matching (deterministic matching) confirmed by first and last names
2. Using simple probabilistic matching using JaroWinkler algorithm (52) (if step 1 fails) or complex algorithms like Fuzzy string matching by Winkler (53) or Levenshtein (54).

Table 2 Further illustrates the circumstances and the values of derivable from the different scenarios.

**Table 2:** Scenarios and implications for patient verification and records linkage.

Scenarios	Circumstance	Operation	Implication
Scenario 1	Both H_1_ and H_2_ are online	H_2_ Registers (generate ID) H_2_ Updates Patient records	H_2_ registers and generates standardized unique ID verifiable on the network. H_1_ downloads the ID.
Scenario 2	H_1_ is online and H_2_ is offline	H_1_ validates Patient identity, retrieve or update records.	H_1_ does not need H_2_ that generated the Patient ID to validate or update a Patient record.
Scenario 3	H_1_ is offline	H_1_ validates Patient Identity and link stale records up to when it went offline.	H_1_ does not need internet access to validate or update a Patient while offline. H_1_ makes use of available encrypted record up to a certain time.

**Figure 5:**
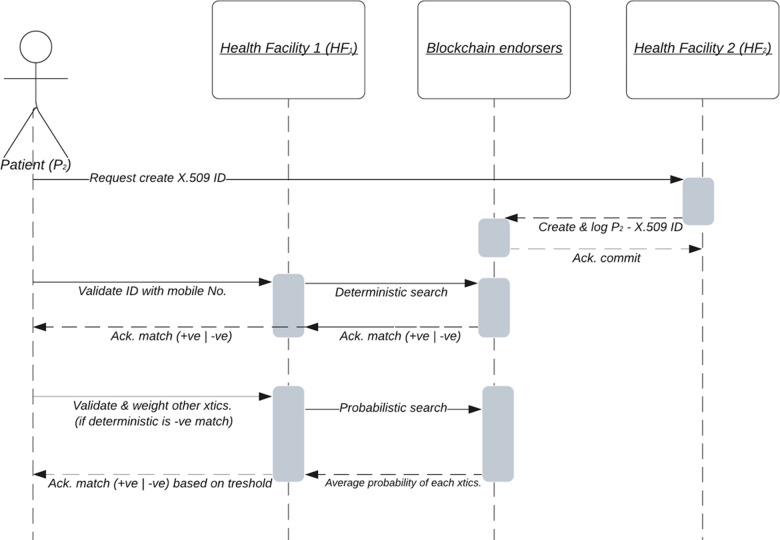
Sequence diagram showing proposed patient ID registration and matching process.

The sequence diagram in Figure 5 shows Patient (P_2_) registered with Health Facility 2 (HF_2_), and aims to access service at Health Facility 1 (HF_1_). Our offline model proposes that health facilities act as identity-generating Certificate Authorities (CA). If identities are generated and logged on the blockchain network or shared EMR, when H_2_ is offline, or if H_1_ is offline but has downloaded the latest block (or shared identity) information with logged identity before going offline, the Patient P_2_ can still be matched. This model is not mutually exclusive of MPI, but can be used either independent or along with MPI to better enhance records matching when authenticating or authorizing institutions are offline. In otherwords, it can facilitate a bottom up approach at enabling futuristic implementation of MPIs in a resource limited setting like the LMICs.

#### Phone number matching step

In quarter-three of the year 2020, the Nigerian government mandated that all mobile phones be linked to the owner's NIN number (55). For many years in Nigeria, telecommunications service providers have been mandated to register phone numbers before activation. Given all the measures, phone-number is emerging as the simplest to remember. Phone number is unique for identifying an individual owner, but not without drawbacks.. The first step uses a deterministic all or none phone number matching as the first step to matching two or more records. For this model to work, we propose a token-based incentive on groups of EHR networks. We assume the EHR networks will be state-networks (sub-regional networks) like our FCT use case for eventual design. A simplified Python implementation of step one is shown in Algorithm 1. The comparison will yield a True or False representing positive-match or negative-match, respectively. As part of the workflow, the health provider will first ask for the phone number. If the deterministic search works, then validate the record with any of the firstName or lastName in the searched record to match at the time of service provision. If a patient has more than one phone number, this implementation will iterate over an array of phone numbers and execute G1 over the phone number array until an exact match is found or no match is found. Deterministic matching comes with its flaws. For instance, a survey of 112 MPIs from 2000 to 2003 found duplicate rates of above 10% for all (50). The ONC report highlighted that this was as high as 39% in a smaller 11 MPI review in another report.

**Algorithm 1:** Deterministically matching records using mobile phone IMEI number

**Input**: Two arrays of one or more phone IMEI records for comparison, Phone_str1 and Phone_str2 (in format “08031234567”)
**Output**: positive-match or negative-match (in format boolean)
# A Simple deterministic matching comparison for phone number records.
G1=(Phone_str1 == Phone_str2)

#### Multi-characteristics matching step

Probabilistic patient matching algorithms vary in their implementation, but they generally use distance (or the minimum number of edits required) of record fields compared. (48, 49) already highlighted how these errors lead to significant deterministic matching variations and mismatches in the US and UK. The fuzzy pattern matching allows for and accommodates significant misspellings and a range of disagreements (e.g., NnaEmeka and Emeka). These misspellings can arise from phonetic misrepresentation (e.g., Emeka and Amaka are both valid names), Typographical errors (e.g., Emka, and Emeka), or morphological confusion (similar character, e.g., lower “L” and capital “I” or “0” and “O”) (27). The relative distance is weighted on a scale of “0” to “1” – “1” being the nearest.

One implementation of Fuzzy string matching is the Python *FuzzyWuzzy* package (56). We here demonstrate our model using this package in Algorithm 2. G2 is used to match the date of birth stored in the form YYYY/MM/DD. If both *firstName* and *lastName* entry form fields in a software solution interface are different, G3a1 and G3a2 are used for matching. Alternatively, if the software solution entry form field uses a single entry field for fullName, then G3b is used to match the full name presented partially. G4 is used to match a patient who has a NIN (with or without a phone number). Algorithm 2 illustrates the implementation of this matching algorithm using Python code. The steps are to match G2, G3, and G4 to determine the match's probability to an existing record. The algorithm will output probabilities ranging between 0 and 1. If the average probability for all compared attributes is above 0.75 (we arbitrarily set this), then it is a positive match. If not, it is a negative match (no match).

**Algorithm 2:** Probabilistic fuzzy matching records using

**Input**: Phone IMEI Phone_str, Patient Characteristics *P*_char={dob, f_name, l_name, nin}
**Output**: true-match, false-match, true-nonmatch, false-nonmatch
# Probabilistic fuzzy matching of patient records.
from fuzzy-wuzzy import fuzzy
G2=fuzzy.ratio(dob1, dob2)
# when names are properly sorted
G3a1=fuzzy.partial_ratio(firstName1.lower(), firstName2.lower()
G3a2=fuzzy.partial_ratio(lastName1.lower(), lastName2.lower()
#when the names are not sorted properly
G3b=fuzzy.token_sort_ratio(fullName1.lower(), fullName2.lower())
#when the Patient had provided their NIN (or international passport no. or drivers license no.)
G4=fuzzy.ratio(nin1, nin2)

#### Generating a decentralized unique ID

In the preceding subsection, we discussed the process of querying, matching, and retrieving a unique Patient's record from a shared health record. For a Patient whose record does not exist in any of the health facility databases, we propose a scheme for generating a standard random but “meaningless” unique 64 digit identifier. This can be useful for Patients who do not have phone numbers or environments where national ID enrolments are not yet widespread. The 64 digit identifier is substring to a readable nine (9) digit human-readable Patient ID, which can be generated offline at any health facility. It can also be provable offline at any other health facility, given the combination of characteristics. For consistent results and better accuracy, all implementation must use all fields entered in string format, and the age field is represented as string digits, not the word equivalent. The gender must be spelled out (e.g., “male” or “female” or “not disclosed”). The spellings for firstName, lastName, and NIN must be accurate for the generated code to be provable at another health facility. When NIN is not available, the healthFacilityID is used temporarily. Our choice of healthFacilityID was to use a mechanism for limiting collision (in case more than one Patient has similar all five characteristics). The healthFacilityID characteristics can be retrieved from a registry encoded as *state_code/lga_code/ward_code/facility_type_code/ownership_code/facility-code* (eg. health facility code can be in form – 01/01/1/1/2/0041).

**Algorithm 3:** Algorithm for offline generation of unique Patient ID (PI)

**Input**: firstName, lastName, age, gender, nin
**Output**: PatientID (PI) - seven-digit characters substringed from Patient characteristics hash # Python implementation of the universally unique Patient ID offline
import hashlib
stringConcat =
firstName.lower()+lastName.lower()+age+gender.lower()+nin.lower()
PatientHash =
hashlib.sha256(stringConcat.encode()).hexdigest()
PatientID=PatientHash[54:-1]

## Discussions

No centralized identification generation and authentication strategy have been proved to meet all the desirable characteristics while remaining cost-effective. Our proposed offline Patient ID generation and matching strategy presented above will contribute to knowledge by helping address this challenge.

### Phone number issues

Phone number used for comparison has a fundamental flaw in that it can change, as users change their phone numbers easily. Besides, it cannot be regarded as completely confidential as it is in the public domain and can be shared without the owner's consent. Many subscribers have more than one phone number in Nigeria and can be re-assigned to another user if not active over time. In order to mitigate against the potential impact of this drawback, a point-based incentive will be used with this algorithm that tracks service uptake of linked phone numbers. When users change the number and do not update their records, they lose points. This will be similar to the strategy used in mobile money wallets. Moreover, during implementation, the nine (9) digit unique Patient ID may be used to replace the phone number for G1 matching.

### Interpreting the matching algorithms

Algorithm 2 described above illustrates how G2 uses deterministic matching within a probabilistic approach. G3 and G4 will complement the match when a match occurs. Any of G2 or G4 and/or G1 or any of G3 will be considered a true match. The weighting will still be documented and used for additional algorithms; for instance, three of (G1, G2, G3, G4) will be 75% matching which is considered a positive match. Nevertheless, two will be 50% matching considered not definite and needs to go to human review. While one (G3 or G4) of the four will yield a 25% match, which is certainly not a match (true negative). When the algorithm matches 50%, which should be rare, it will be manually human-checked for early implementation, and each hospital's system will learn the subsequent feedback for future match improvements.

### Using the model in practice

This model can be used in many ways starting from the FCT EMR network system. The first step would be to replace (or generate a parallel Patient ID) the existing serially generated ID with a Patient ID. Digital health vendors can set up a shared record blockchain for sharing frequently requested Patient shared-health records. Hospitals can upload these anonymized records onto the blockchain network when they are online. Patients would have to individually opt-in for the specific fragment of the shared health records to be uploaded to the blockchain for their access or their physician's access. The receiving health facility will then be able to check and match patient records in the event of intermittent or no network without requiring network availability at sending institution. Various models of Blockchain-based shared records have been documented here (57).

**Figure 6:**
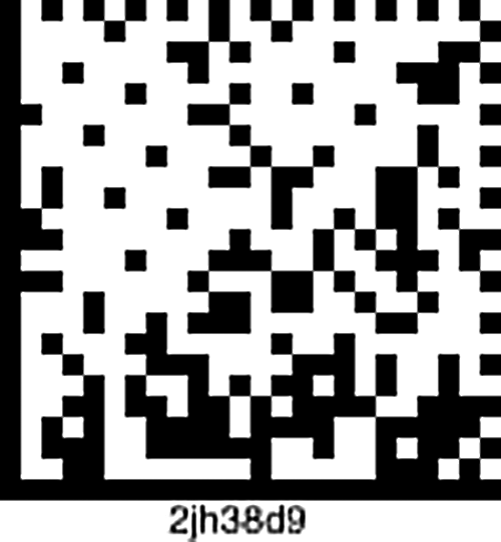
Sample data matrix containing encoded patient data.

Using this model will also eliminate the need for a central authentication authority to validate Patients. So far, the model meets three of the four characteristics of a decentralized ID. Our Patient ID scheme meets the first three characteristics of a decentralized ID required by the W3C proposed standard. The ability to discover metadata can happen in either of two ways. One is making metadata available on the blockchain. This comes with additional network overhead for implementing health facilities. Alternate implementations can also encode a Patient's data onto a data matrix, as seen in the sample implemented using Python in Algorithm 4. The generated data matrix is seen in Figure 6. The encoded data can be read using an appropriate data matrix decoder. Though these implementations used Python, they can easily use any other programing language. Also, this model can easily be extended to X.509 certificates for identity management as in our sequence diagram.

**Algorithm 4:** Algorithm for offline generation of a unique Patient data matrix

**Input**: firstName, lastName, age, gender, phoneNumber
**Output**: Patient Data Matrix – An image of the Patient data subset
# Python implementation of data matrix generator with the Patient data
#Please install libdmtx and pillow python libraries
from pylibdmtx.pylibdmtx import encode as enc
from PIL import Image
encoded=enc("Emeka Chukwu-08012345678-30yrs-Male")
img=Image.frombytes("RGB", (encoded.width, encoded.height), encoded.pixels)
img.save('dmtx.png')

In practice, there are some data cleaning and formatting necessary before applying these algorithms in production. All Python codes in our Algorithms 1–4 are for test purposes and should be used with care as they are only illustrative. It is best practice to use cryptographic encryption to store and retrieve sensitive Patient information (or Protected Health Information), preferably pre-encoded in Fast Healthcare Interoperability Resource (FHIR) format (58). While there are many hashing algorithms, our proposed hashing algorithm is SHA256 for consistency and security. When used at scale, this can help sub-regional and national governments transition from aggregate-based data collection and use to individualized and integrated Patient data collection and use.

### Generalizing the model

The Nigerian use case inspired this proposed model; however, it is generalizable as the key artifacts and algorithms can be used in other jurisdictions.

### Limitations

A fundamental limitation of our proposed model is that we assume that Patients will not want to change their phone because of incentives on the line. We acknowledge that in practice, some patients may lose their phone or decide to willfully change their phone, thus resulting in duplicate records.

Also, our work acknowledges that clinician burnout is real, as illustrated in a recent study (59). However, we mitigate against this by ensuring that Patients are asked their number, and if a positive first match is returned, the Patient is only asked any of their first or last names to validate the returned record.

Another limitation of our work is that we did not simulate the data to determine its performance; however, this un-simulated work will still prove invaluable and revolutionary in low and middle-income countries.

## Conclusion

Patient identification remains a wicked problem for many health systems. Using the Nigerian context, we have presented a case for digital unique patient identification and the available options with their strengths and drawbacks. The design of a dual deterministic-probabilistic matching algorithm was also proposed and demonstrated. We presented a simplistic Python algorithm-based code for this model. We also implemented an algorithm for a universal offline unique Patient ID (PI) generation and provability. We show that this model meets the four characteristics of a decentralized ID: no central authority, cryptographically provable, metadata discoverable, and outlive issuing institution. This model will help lay the groundwork for scale-up and fast-track a cost-effective implementation of MPIs for jurisdictions where governance is more centralized or when the infrastructure to support MPI becomes mature. We further posit that this model will enable attainment of UHC in LMICs by eliminating double counting through de-duplication of healthcare data and in turn ensuring accuracy in monitoring and evaluating effectiveness of health care programs and services. It will also improve care coordination, data privacy and seamless exchange of patient health records. Our future work will include implementing this algorithm in health facilities to test this new model's hypothesis. In the future, we will work with relevant digital health stakeholders to determine the optimal set of Patient characteristics that will reduce discrimination while ensuring the most significant number of Patients can have unique ID generated and validated offline. In the future, the model will be extended to support the X.509 certificate standard.

## Data Availability

The original contributions presented in the study are included in the article/**Supplementary Material**, further inquiries can be directed to the corresponding author/s.
