# Supplementary material for: Scaling up a decentralized offline patient ID generation and matching algorithm to accelerate universal health coverage: Insights from a literature review and health facility survey in Nigeria
Source: Front Digit Health. 2022 Sep 7;4:985337. doi: 10.3389/fdgth.2022.985337 (PMC9489848; doi:10.3389/fdgth.2022.985337)
Supplement: Supplementary file 1 [file Data_Sheet_1_v1.pdf]

QUESTIONNAIRE FOR ASSESSMENT OF PATIENT IDENTIFICATION NUMBER IN HEALTH FACILITIES

Date:___________________ Name of Health Facility: _________________________________

Facility Type (Please circle as appropriate): Public Private Primary Secondary Tertiary

Dear Medical Records Officer,
The HHSS eHealth Unit would like to know how patient identification numbers are generated and assigned in your facility. We would like to have your honest feedback. We want to assure you that your particpation is optional and confidential, so you will not be identified as a particpant. This should take about 2-3 minutes of your time. Would you like to help us?

Please carefully read and answer all the questions below (kindly tick as applicable)

1. How do you generate or assign patient ID?
a) Manual
b) Computer
c) Others (please specify)

2. What format is the ID?
a) Numbers
b) Letters
c) Combination of both numbers and letters
d) Others (please specify)

3. How many digits is the ID?
a) Less than 5
b) 5-10
c) Greater than 5

4. Is the ID serial or random?
a) Serial
b) Random
c) Don't know

5. Which of the following ID Standards do you also capture?
a) Phone Number
b) National ID Number (NIN)
c) BVN
d) None
e) Don't know

6. Example of your ID ……………………………………………….
